# Phagocytosis Is the Main CR3-Mediated Function Affected by the Lupus-Associated Variant of CD11b in Human Myeloid Cells

**DOI:** 10.1371/journal.pone.0057082

**Published:** 2013-02-22

**Authors:** Liliane Fossati-Jimack, Guang Sheng Ling, Andrea Cortini, Marta Szajna, Talat H. Malik, Jacqueline U. McDonald, Matthew C. Pickering, H. Terence Cook, Philip R. Taylor, Marina Botto

**Affiliations:** 1 Centre for Complement and Inflammation Research, Division of Immunology and Inflammation, Department of Medicine, Imperial College, London, United Kingdom; 2 Cardiff Institute of Infection and Immunity, Cardiff University School of Medicine, Cardiff, United Kingdom; Keio University School of Medicine, Japan

## Abstract

The CD11b/CD18 integrin (complement receptor 3, CR3) is a surface receptor on monocytes, neutrophils, macrophages and dendritic cells that plays a crucial role in several immunological processes including leukocyte extravasation and phagocytosis. The minor allele of a non-synonymous CR3 polymorphism (rs1143679, conversation of arginine to histidine at position 77: R77H) represents one of the strongest genetic risk factor in human systemic lupus erythematosus, with heterozygosity (77R/H) being the most common disease associated genotype. Homozygosity for the 77H allele has been reported to reduce adhesion and phagocytosis in human monocytes and monocyte-derived macrophages, respectively, without affecting surface expression of CD11b. Herein we comprehensively assessed the influence of R77H on different CR3-mediated activities in monocytes, neutrophils, macrophages and dendritic cells. R77H did not alter surface expression of CD11b including its active form in any of these cell types. Using two different iC3b-coated targets we found that the uptake by heterozygous 77R/H macrophages, monocytes and neutrophils was significantly reduced compared to 77R/R cells. Allele-specific transduced immortalized macrophage cell lines demonstrated that the minor allele, 77H, was responsible for the impaired phagocytosis. R77H did not affect neutrophil adhesion, neutrophil transmigration *in vivo* or Toll-like receptor 7/8-mediated cytokine release by monocytes or dendritic cells with or without CR3 pre-engagement by iC3b-coated targets. Our findings demonstrate that the reduction in CR3-mediated phagocytosis associated with the 77H CD11b variant is not macrophage-restricted but demonstrable in other CR3-expressing professional phagocytic cells. The association between 77H and susceptibility to systemic lupus erythematosus most likely relates to impaired waste disposal, a key component of lupus pathogenesis.

## Introduction

Complement receptor type 3 (CR3, also known as Mac-1, CD11b/CD18, αMβ2) is a heterodimeric transmembrane receptor found on most immune cells including dendritic cells (DCs), monocytes/macrophages, neutrophils and NK cells. A wide range of ligands have been described for CR3, including complement activation fragments (C3b/iC3b) [1], intravascular adhesion molecule-1 (ICAM-1, CD154 [2]), fibrinogen [3], high mobility group box protein 1 (HMGB-1) [4] and lipopolysaccharide (LPS) [5]. CR3 has been shown to contribute to cell activation, chemotaxis, cytotoxicity, phagocytosis [6], [7] and tolerance induction [8].

Genome-wide association studies (GWAS) have shown that an allelic variant of the alpha-chain, encoded by the *ITGAM* gene, is associated with risk of developing systemic lupus erythematosus (SLE) [9]–[11]. The strongest association between *ITGAM* and risk of SLE is with the minor allele of a non-synonymous SNP, rs1143679 (odds ratio 1.4–2.17), which converts the arginine at amino acid position 77 to a histidine (R77H, minor allele frequency of ∼10% in European American individuals) [9]–[11]. This variant does not appear to increase the risk for other autoimmune conditions, except for systemic sclerosis [12], [13], for which the association is much weaker [14]. Although the possibility of other independent rare causal variant(s) within the *ITGAM* gene cannot be ruled out with certainty, imputation-based association results have confirmed that rs1143679 remains the most promising candidate for causal association with SLE [13]. The rs1143679 SNP encodes the Mart alloantigen that can cause alloimmune neutropenia in neonates [15]. Interestingly some anti-Mart antibodies are able to interfere with Mac-1-dependent adhesive properties of neutrophils and monocytes and to prime neutrophils for the production of reactive oxygen species [15].

Structurally CD11b consists of five extracellular domains and a small cytoplasmic domain. The extracellular part of the protein is composed of seven 60 amino acid repeats that fold into a seven bladed beta-propeller and an inserted (I) domain of 200 amino acids between beta-sheets 2 and 3 of the beta-propeller. Ligand binding appears to take place in the I domain [16]. The R77H polymorphism is within the beta-propeller domain and currently it is unclear how it may affect ligand binding, particularly as the full crystal structure of CR3 has not yet been resolved.

Two recent studies have reported that the lupus-associated minor CD11b allele (77H) impairs phagocytosis and adhesion [17], [18]. The first study used only transfected cell lines expressing the 77H and 77R variants, whilst the second study also analysed *ex-vivo* human 77H/H cells and demonstrated functional perturbations in monocytes/macrophages carrying the SLE-associated allele. To define the *in vivo* significance of R77H we elected to study the risk allele in heterozygosity as this is the genotype of the vast majority of SLE patients [9]–[11] and to assess several CR3-mediated activities in the major human CR3 expressing cell types: monocytes, macrophages, neutrophils and DCs. Using this systematic and comprehensive approach we found that the lupus-associated 77H allele impairs the phagocytosis of iC3b-coated particles but does not appear to affect other CR3-mediated functions including neutrophil adhesion and *in vivo* transmigration. CR3 activation through iC3b-coated targets inhibited to a certain degree the TLR7/8-mediated pro-inflammatory cytokine release by 77R/H monocytes but, unlike the report by Rhodes et al [18] with 77H/H monocytes, this effect was not influenced by R77H. Our data demonstrate that the 77H allele selectively influences CR3-mediated phagocytosis. The removal of cell debris (waste disposal) without causing either inflammation or triggering an autoimmune response is an important physiological activity. Abnormalities in waste disposal pathways have been associated with lupus pathogenesis [19], [20]. The robust genetic association between the 77H allele and lupus susceptibility together with our data demonstrating a reduction in phagocytosis by 77R/H-expressing phagocytic cells strengthen the hypothesis that abnormalities in waste disposal pathways play a key role in lupus pathogenesis.

## Materials and Methods

### Donors and samples

Peripheral blood samples were obtained by venepuncture of healthy adult volunteers after obtaining informed written consent in accordance with the Declaration of Helsinki. The study was approved by the Hammersmith and Queen Charlotte's & Chelsea Research Ethics Committee (REC 2000/6000). DNA was obtained using a buccal kit according to the manufacturer instruction (Qiagen Ltd, UK). The rs1143679 variant of *ITGAM* was genotyped by direct sequencing of the PCR amplicon (forward primer: ^5′^AGTGCGACTACAGCACAGGCTCAT^3′^ and reverse: ^5′^GAGACAAGGAGGTCTGACGGTGAA^3′^ ). All the volunteers were healthy with no history of autoimmune conditions.

### Human cell isolation

All blood samples were treated in a similar manner and processed promptly. All *ex-vivo* assays were performed using freshly isolated cells from sex- and ethnicity-matched individuals, one individual homozygous for the common allele (77R/R) paired with one 77H/H or 77R/H subject. Human neutrophils (PMNs) were isolated by dextran sedimentation and discontinuous plasma-Optiprep gradients [21] followed by negative selection using a custom antibody cocktail containing antibodies to CD36, CD2, CD3, CD19, CD56, glycophorin A and beads (Stemcell Technologies, Vancouver, Canada) as previously described [22]. Cell purity was consistently >95% as verified by flow cytometry (CD16^hi^CD14^neg^CD11b^+^) and cytospin. Monocytes were obtained by density gradient separation coupled with a negative selection kit for human monocytes as recommended by the manufacturer (Miltenyi Biotec GmbH, Germany). Monocyte-derived macrophages (for simplicity abbreviated as macrophages) were generated by culturing the cells for two days in RPMI-1640 medium supplemented with 10% fetal calf serum (FCS), 2 mM L-glutamine, 1% penicillin/streptomycin (Life Technologies, Grand Island, NY) and 20 ng/ml recombinant human macrophage colony stimulating factor (M-CSF) (PeproTech, Rocky Hill, NJ) as described previously [23]. Monocyte-derived dendritic cells (DCs) were generated by culturing monocytes for 5–6 days in RPMI 1640 medium, 10% FCS, 1% penicillin/streptomycin, 2 mM L-glutamine, 100 ng/ml recombinant human granulocyte-macrophage colony stimulating factor (GM-CSF) (PeproTech) and 50 ng/ml recombinant human IL-4 (PeproTech). Every 2–3 days, 50% of the culture media was exchanged with fresh media.

### Murine cells

C57BL/6 mice were obtained from Harlan UK. CD11b-deficient mice (*Itgam* ^−/−^) [24] were from Jackson Laboratory (Bar Harbor, ME, USA) and were backcrossed on the C57BL/6 genetic background for 10 generations. All animals were handled in accordance with institutional guidelines and procedures approved by the UK Home Office in accordance with the Animals (Scientific Procedures) Act 1986. Conditional-Hoxb8 immortalized myeloid precursors were generated from C57BL/6 and *Itgam* ^−/−^ mice as previously described using a retrovirus encoding an estrogen receptor binding domain Hoxb8 fusion protein [25]. The *Itgam* ^−/−^ myeloid precursors were further transduced with pMXs-IZ vector containing the human CD11b variants. The cDNA of the 77R CD11b allele was obtained from OriGene Technologies (Rockville, MD, USA, SC315229, NM-000632.2), whilst the lupus susceptible allele (77H) was obtained by site-directed mutagenesis by changing the G at position 230 to an A (R77H). Both alleles were checked by sequencing. As control an empty vector was added to conditional-Hoxb8 immortalized myeloid precursors generated from C57BL/6 *Itgam* ^−/−^ cells. The different Hoxb8 immortalized myeloid precursors were differentiated *in vitro* into murine neutrophils or macrophage cell lines by removing estrogen and resuspending the cells in the appropriate differentiation medium [Opti-MEM, 10% v/v FCS, 2 mM L-Glutamine, 1% Penicillin/Streptomycin and 30 µM-mercaptoethanol supplemented with 20 ng/ml recombinant murine stem cell factor (SCF) (PeproTech) and recombinant murine granulocyte colony stimulating factor (G-CSF) (PeproTech) for the neutrophils; RPMI 1640, 10% v/v FCS, 2 mM L-Glutamine, Penicillin/Streptomycin, and 20 ng/ml recombinant murine M-CSF (PeproTech) for the macrophages] as previously reported [25], [26]. By day 4, neutrophils routinely represented ∼98% of the cells and were identified by flow cytometry as Ly-6B.2^high^CD117^low^ cells and on cytospins by their typical nuclear morphology. By day 5, 90–98% of the macrophages expressed high levels of F4/80 [27]. Cell surface expression of the heterodimer (murine CD18/human CD11b) was assessed by flow cytometry using the following antibodies: anti-human CD11b (ICRF44) or anti-mouse CD11b (M1–70), the latter cross-reacts with human CD11b.

### Flow cytometry

Murine and human leukocytes were stained using standard protocols in the presence of a saturating concentration of 2.4G2 mAb. The following antibodies were used: anti-mouse CD11b (M1–70), anti-mouse Ly-6B.2 (7/4), anti-mouse CD117 (2B8), anti-mouse F4/80 (BM8), anti-human CD11b (ICRF44 and D12), anti-human CD11b (active epitope) (CBRM1/5), anti-human CD14 (61D3), anti-human CD16 (3G8), anti-human CD62L (DREG56). Antibodies were purchased from BD Biosciences Pharmingen (San Diego, CA) or eBioscience (San Diego, CA). In some assays PMNs were stimulated with 25 nM phorbol myristate acetate (PMA; Sigma) for 5, 10 and 15 min prior to the staining. Data were acquired using a FACSCalibur (Becton-Dickinson, Mountain View, CA) and analyzed using FlowJo software, version 7.6 (TreeStar, Ashland, OR).

### Phagocytosis assays

CD11b-mediated phagocytosis was performed using human and murine cells and using three types of iC3b-coated particles: mouse apoptotic thymocytes, guinea pig red blood cells (gRBCs) and fluoresbrite® carboxylate YG 1.5 µm microspheres. Apoptotic thymocytes and gRBCs (TCS Bioscences, Buckingham, UK) were washed three times in PBS/1%BSA and then resuspended to 1%v/v in PBS. These cell suspensions were incubated with pHrodo™ Dye Succinimidyl Ester (1 µg/ml, Life Technologies, Grand Island, NY) for 30 minutes at room temperature. After three washes the cells were opsonised with mouse C5-deficient serum at 37°C for 30 mins (gRBCs-miC3b) and resuspended to 1%v/v in culture medium. Fluoresbrite® carboxylate microspheres (Polysciences Inc, Warrington, PA) were resuspended (1/200) in Krebs' Ringers PBS-Glucose buffer with human iC3b (20 µg/ml, Complement Technology Inc, USA) and incubated at 37°C for 30 mins. Human iC3b-coated beads were then washed with PBS/1%BSA/EDTA and resuspended in culture medium (1/400). For all experimental protocols the level of iC3b-opsonisation was checked by flow cytometry using a biotinylated polyclonal antibody that recognises human and mouse C3 fragments (Clone: 6C9, Cedarlane Labs, Ontario, Canada) followed by streptavidin-PE (BD Biosciences Pharmingen). In each phagocytic assay complement-opsonised particles were fed at a 10∶1 ratio to DCs (0.7×10^5^ cells/well), macrophages (1×10^5^cells/well), PMNs (2×10^5^cells/well) or monocytes (2×10^5^cells/well) in U-bottom wells. Cells were collected at different time points as indicated in the result section and the uptake quantified by flow cytometry. Phagocytosis was calculated as % of cells that had internalised the iC3b-coated particles. Non-opsonised particles were used as negative controls. In selective experiments phagocytosis was visualised using ImageStream (Amnis, Seattle, WA). The data obtained were analyzed using the ImageStream Data Exploration and Analysis Software (IDEAS, Amnis). Single focused cells were gated by area aspect ratio followed by gradient root mean square of the brightfield image.

### PMN – functional assays

Freshly isolated PMNs were assessed in several functional assays:

- **Rosetting.** Guinea pig RBCs were labelled with carboxyfluorescein diacetatesuccinimidyl ester (CFSE) as described previously [28]. Different amounts of mouse or human iC3b was deposited on the gRBCs by using increasing dilution of mouse or human C5-deficient serum respectively. Freshly isolated PMNs were washed and resupended in 1 mM Mg^2+^/1 mM Ca^2+^ buffer and incubated with gRBCs-iC3b for 30 min at 4°C with gentle shaking. Rosetting was assessed by flow cytometry and on slide (at least 200 PMNs analysed). PMN binding ≥3 RBCs was recorded as a single rosette.
- **Adhesion assays.** Adhesion was measured by coating Immunolon plates with fibrinogen (10 µg/ml). Plates were blocked with PBS 1% BSA for 3 hr at room temperature. Freshly isolated PMNs were then added (2×10^5^cells/well) and incubated for 90 min at 37°C. Cells were gently washed with Opti-MEM medium and adhesion assessed by staining the adherent cells with crystal violet.
- **Oxidative burst.** Production of reactive oxygen species (ROS) was studied by loading PMNs (10^5^cells/ml) with 2′, 7′-dichlorodihydrofluorescin diacetate (DCFH-DA) (Sigma) (5 µM) for 30 min at 37°C. After stimulation with PMA (25 nM) for 30 min at 37°C, the increase in ROS production was measured by flow cytometry.
- **Neutrophil extracellular traps (NETs).** PMNs seeded at 10^5^cells/well in complete medium (DMEM 10% v/v FCS, 2 mM L-Glutamine, Penicillin/Streptomycin without phenol red) were stimulated with 25 nM PMA (final concentration) for 3 hours. Quantification was performed as described previously [29].
- **In vitro chemotaxis assay.** Chemotaxis assays were performed as before [25]. Briefly, transwells (3 µm polycarbonate membrane, 6.5-mm-diameter wells; Costar, Corning, NY, USA), pre-treated with 2% (w/v) gelatin (porcine skin type A; Sigma) were seeded with mouse microvascular endothelial cells (3×10^4^/transwell) (kindly donated by Prof F. Marelli-Berg, Imperial College London) [30] or human umbilical vein endothelial cells (HUVEC) (3×10^4^/transwell). After overnight culture, transwells were washed, recombinant murine macrophage inflammatory protein (MIP)-2 (PeproTech) was added to the bottom chamber at a final concentration of 1 nM and 5×10^5^ neutrophils (murine neutrophil cell lines or freshly isolated human PMNs) were added to the top chamber. At different time points (from 30 to 120 minutes) cells were collected from the bottom chamber for analysis. The number and the viability of migrated cells were assessed by flow cytometry by adding counting beads (Life Technologies) and by staining the cells with propidium iodide.
- **In vivo adoptive transfer model.** *In-vitro*-differentiated *Itgam* ^−/−^ murine neutrophils reconstituted with one of the human CD11b variants (mCD18/hCD11b-77R or mCD18/hCD11b-77H) were labelled with either 1 µM Cell Trace dodecyldimethylamine oxide succinimidyl ester (DDAO) (Life Technologies) or 250 nM CFSE (Life Technologies). DDAO- and CFSE-labelled neutrophils (5×10^6^ of each type) were mixed in a 1∶1 ratio in PBS and injected i.v. into C57BL/6 mice. This was immediately followed by an i.p. injection of 4% thioglycollate broth (0.4 ml/mice, Sigma) or 330 nM MIP-2 (0.2 ml/mice, PeproTech). PBS injection was used as control. Two hrs after MIP-2 injection and 4 hours after the thioglycollate injection, the mice were sacrificed and the peritoneal cells were recovered by lavage with 5 ml of cold 5 mM EDTA in HBSS. Total cell counts and differential analyses were performed by flow cytometry.

### Cytokine assays

Monocytes (2×10^5^ cells/well) and DCs (0.7×10^5^cells/well) were incubated with iC3b-coated particles alone (gRBCs-miC3b or hiC3b-coated beads). Non-opsonised particles and medium were used as controls. In separate wells cells were pre-incubated for 1 hr with hiC3b-coated beads and then stimulated with R848 (Life Technologies) (2 µg/ml or 10 µg/ml accordingly to the cell type). Supernatants were collected after 24 hrs and frozen until analysis. Cytokine levels were measured with a bead multiplex assay (eBioscience) according to the manufacturer's instructions. IL-1β, IL-6, IL-10, IP-10, TNF-α were measured. Results were analysed in two ways: i) the difference in cytokine production (expressed as absolute value) between samples pre-incubated with coated particles to samples pre-incubated with uncoated particle or medium (as indicated in the figure legends) and ii) as % of change induced by iC3b coated particle compared to medium or uncoated particle. Both analyses gave statistically similar findings.

### Statistics

Data are expressed as mean+/−SEM. Statistical analysis was performed using GraphPad Prism version 3.0 (GraphPad Software, San Diego, CA). Unless otherwise stated, data from *in vitro* assays were analysed by two-tailed Student's t-test for paired samples. One-way analysis of variance with Bonferroni's multiple comparison tests were applied for analysis of multiple groups. Differences were considered significant for p values<0.05.

## Results

### The lupus CD11b variant (77H) does not affect CD11b cell surface expression

Due to the rarity of the minor allele (77H), the vast majority of SLE patients are heterozygous (77R/H) and not homozygous for the risk allele [11]. As a consequence, we elected to compare healthy subjects that were homozygous for the non-risk allele (77R/R) with healthy individuals carrying the risk allele in heterozygosity (77R/H). However, for each assay we also extended the analysis to three individuals that were homozygous for the risk allele (77H/H). We did not detect any obvious differences in these assays between 77R/H and the three 77H/H samples. Therefore in representing our data we pooled the findings obtained from the homozygous 77H/H and heterozygous 77R/H individuals (77R/H-77H/H).

**Figure 1:**
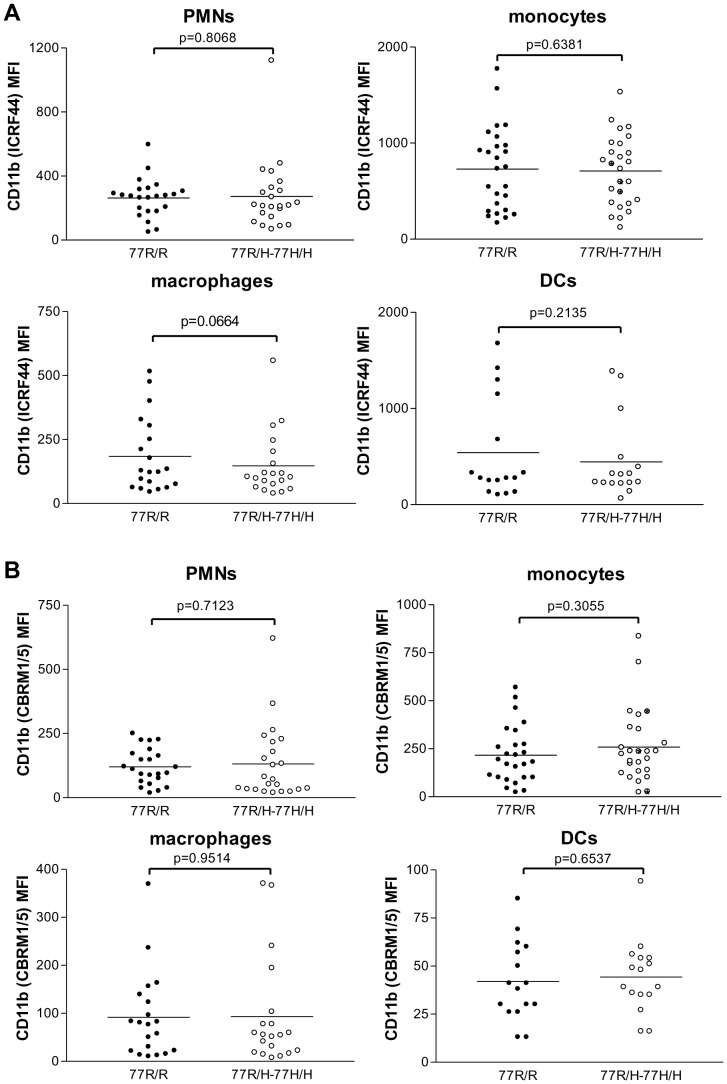
Cell surface expression of CD11b on different cell populations. The expression was quantified by flow cytometry using ICRF44 (A) and CBRM1/5 (B) antibodies. The latter only recognises the headpiece of CD11b in its active state. Data are presented in mean fluorescence intensity (MFI), closed symbols 77R/R donors, open symbols 77R/H-77H/H donors. The two groups were not statistically different. Bars indicate means.

We initially assessed whether the 77H variant influenced CD11b cell surface expression on resting cells. We measured CD11b expression on PMNs, monocytes, macrophages and DCs by flow cytometry using the ICRF44 antibody. Though the CD11b expression varied among individuals there was no genotype-specific difference (figure 1A). Similarly the analysis of the active high affinity state of CD11b with the CBRM1/5 antibody failed to reveal any significant difference between the 77R/H and the 77R individuals (figure 1B). We also assessed how quickly the two CD11b variants changed conformational state in response to stimuli by performing a time course experiment with PMNs activated with PMA (25 nM). The response of 77R/H PMNs was similar to that of 77R/R PMNs (figure S1).

### The CD11b risk allele leads to a defective uptake of complement-coated particles without altering the binding affinity for iC3b

**Figure 2:**
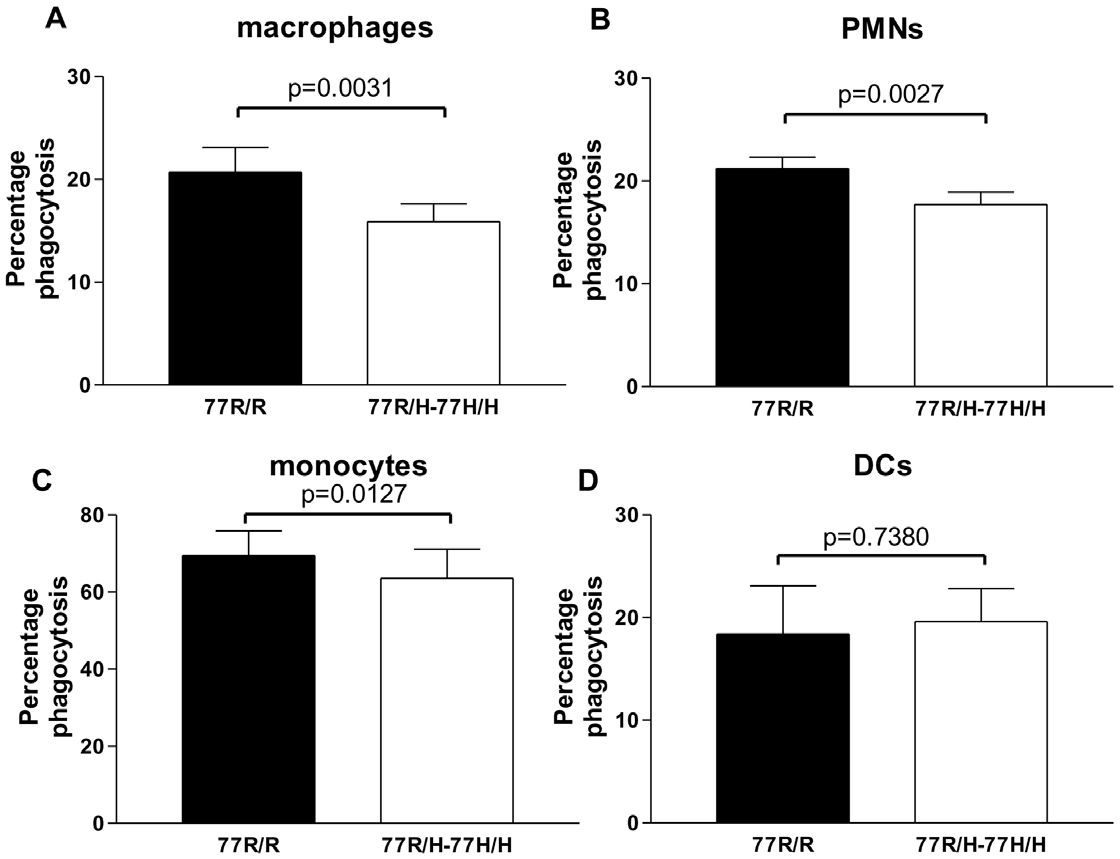
Phagocytosis of hiC3b-coated fluorescent beads. The uptake by macrophages (A), PMNs (B), monocytes (C) and DCs (D) was quantified by flow cytometry and the data are represented as percentage of phagocytosis. Data are expressed as mean+/−SEM. Filled columns 77R/R individuals, open columns 77R/H-77H/H individuals. Paired T test was applied and the p values are indicated.

CR3 is known to mediate phagocytosis of complement opsonised microorganisms. We investigated whether the SLE-associated allele affected this CR3-mediated function using two different particles: gRBCs-miC3b and hiC3b-coated beads. The use of gRBCs opsonised with mouse iC3b is likely to reduce the strength of the receptor-ligand interaction allowing the detection of subtle abnormalities, whilst the use of hiC3b-coated beads minimises the confounding contribution of other phagocytic mechanisms as the particles do not have other molecules expressed on their surface and cannot release intracellular components. gRBCs-miC3b were labelled with pHrodo that changes colour when the particles are fused with the lysosome allowing us to selectively quantify engulfed particles. In the assay with hiC3b-coated bead ImageStream was used to confirm that the vast majority (approximately 98%) of the positive cells had engulfed at least one bead (data not shown). Using both iC3b-coated particles we found that phagocytosis by 77R/H cells (macrophages, PMNs and monocytes) was significantly less compared to the uptake by the corresponding 77R/R cells (figure 2 and figure S2). The mean values were statistically significant if the data were analysed as either as percentage phagocytosis (figure 2) or as percentage difference to 77R/R cells standardised to 100% in each assay (macrophages after 30 min: 77R/R 100 vs 77R/H-77H/H 80.5+/−5.8, p = 0.0054, 13 pairs; PMN after 15 minutes: 77R/R 100 vs 77R/H-77H/H 83.9+/−4.5, p = 0.0039, 13 pairs; monocytes after 18 hrs: 77R/R 100 vs 77R/H-77H/H 90.0+/−3.6, p = 0.0315, 7 pairs). However, no genotype-specific defect in phagocytosis was detected using DCs (% of phagocytosis after 1 hour: 100 in 77R/R vs 161.0+/−28.0 in 77R/H-77H/H individuals, p = 0.0608, 9 pairs).

**Figure 3:**
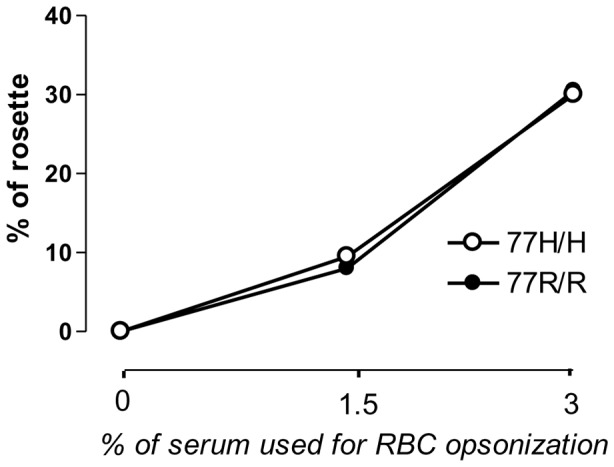
Rosetting assay. Percentage of rosettes formed by CFSE-labelled RBC-hiC3b with freshly isolated PMNs from donors carrying the susceptible allele (77H/H, open symbol) or the common allele (77R/R, closed symbol). One representative assay out of 3 independent experiments.

Although *in silico* models indicate that the iC3b binding takes place in the I domain and the 77R/H polymorphism is unlikely to affect this binding site, we explored whether the differences observed in phagocytosis were the result of different binding to CR3. To address this issue in the most physiological *ex-vivo* setting we used the traditional rosetting binding assay. Using this assay we confirmed that the R77H polymorphism does not alter the binding affinity for iC3b, at least on PMNs (figure 3). We also analysed the adhesion of 77R/R and 77R/H-77H/H PMNs to plates coated with fibrinogen and detected no differences between the two genotypes (data not shown).

To circumvent all the technical limitations of using *ex-vivo* human cells and to confirm that the impaired phagocytosis was indeed specific to the R77H polymorphism and not due to another independent variant(s) within the *ITGAM* gene or a gene closely linked (e.g. *ITGAX*), we generated conditional-Hoxb8 immortalized myeloid precursors from CD11b-deficient mice and transduced them with either the 77R or 77H human CD11b variant. The CD11b-transfected precursors were then matured into fully differentiated macrophages or PMNs using the appropriate conditional media [25], [27]. Importantly, the immortalized cell lines had similar cell surface expression of the hybrid CR3 molecule (mCD18/hCD11b-77R or mCD18/hCD11b-77H) (figure S3A). We then performed the same phagocytic and binding assays applied to the human cells. The uptake of pHrodo loaded gRBCs-miC3b by (mCD18/hCD11b-77H)-expressing macrophages was significantly reduced compared to the engulfment by (mCD18/hCD11b-77R)-expressing macrophages (p = 0.0256), mirroring the data obtained with the human cells (figure S3B). A similar phagocytic defect was observed using opsonised apoptotic cells, whilst the clearance of non-opsonised cells was not affected by the expression of CD11b suggesting the engagement of other receptors (figure S3C). Consistent with the findings with *ex-vivo* human PMNs no differences were detected in the percentage of gRBC-hiC3b bound to the immortalised neutrophil cell lines expressing the hybrid CR3 molecules (figure S3D).

### In vivo and in vitro migration of neutrophils is not affected by the 77R/H CD11b polymorphism

**Figure 4:**
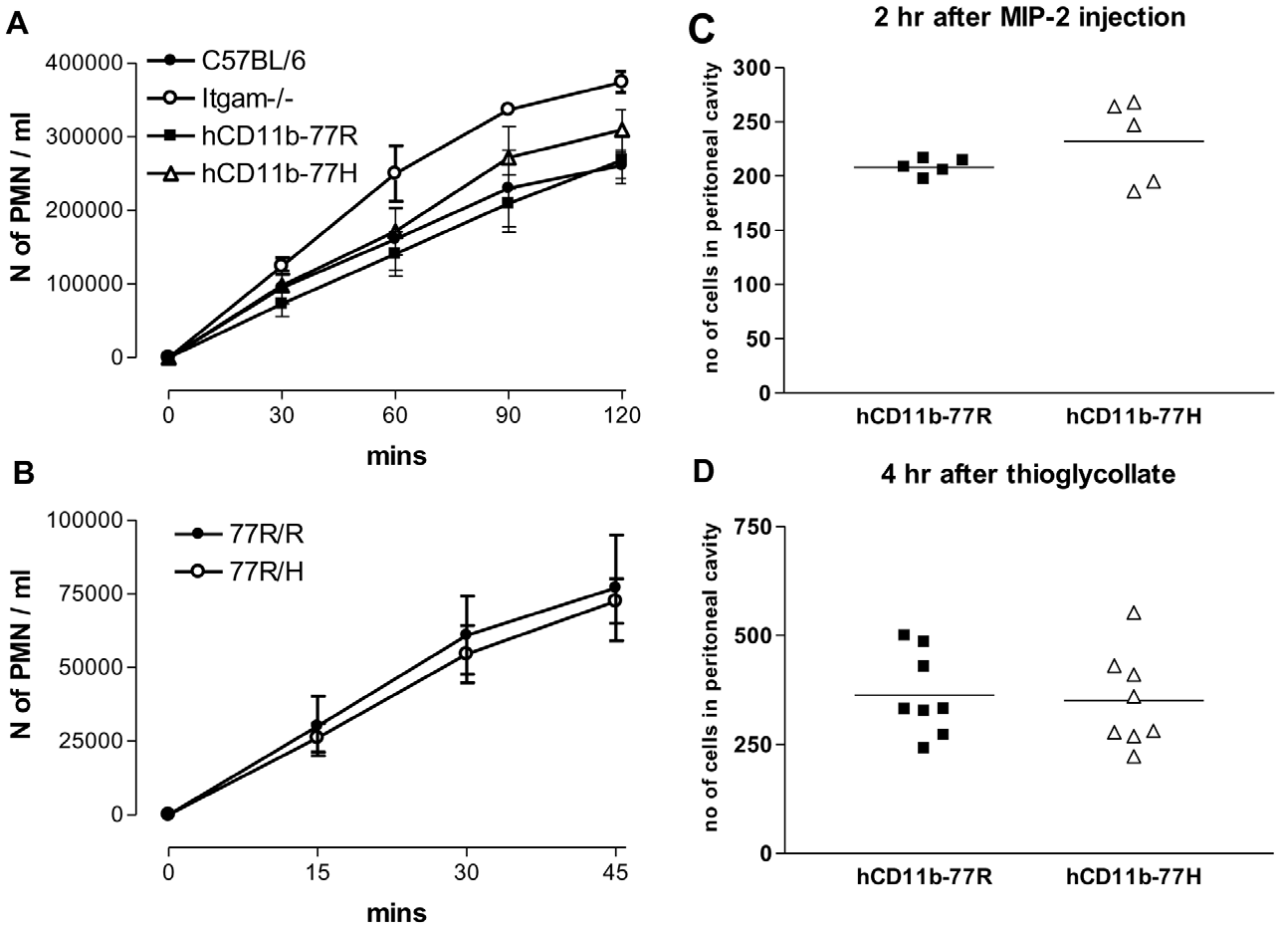
*In vitro* and *in vivo* PMN migration. (A) Migration of neutrophil cell lines through transwells seeded with mouse endothelial cells. PMNs migrated into the bottom chamber in response to MIP-2 were counted at different time points as indicated. Pooled results from at least 4 independent experiments are presented as mean ± SEM. *Itgam*−/− and wild type C57BL/6 neutrophil cell lines were used as controls. CD11b-deficient PMNs, known to have weaker endothelial interactions, migrated faster than the C57BL/6 and the hCD11b expressing cell lines (p<0.001 at 60 mns and p<0.05 at 90 mns). Statistical analysis by Bonferroni's multiple comparison test. (B) Time course of the migration of freshly isolated human 77R/R or 77R/H neutrophils through a HUVEC layer in response to MIP-2. Pooled results from at least 4 independent experiments are presented as mean ± SEM. (C, D) In vivo peritoneal migration of hCD11b-77R and hCD11b-77H PMN cells lines following i.p. injection of MIP-2 (C) and thioglycollate (D). The two hCD11b expressing PMN lines were labelled with DDAO or CFSE and adoptive transferred at a 1∶1 ratio into C57BL/6 mice. Absolute numbers of labelled PMNs recovered from the peritoneum are shown. Data of one out of at least 3 independent experiments are presented. Bars indicate means.

Given the technical hurdle of comparing resting neutrophils isolated from different individuals we elected to use the immortalised neutrophil cell lines to investigate whether the 77H variant altered the ability of PMNs to migrate through an endothelium layer. We initially measured the migration through a mouse endothelium layer in response to MIP-2 using a transwell assay and found no differences between the CD11b-deficient *in vitro*-derived neutrophils reconstituted with either of the two hybrid CR3 molecules (figure 4A). As previously reported [25], the CD11b-deficient PMNs, used as negative controls, showed an accelerated migration compared to the wild type PMNs. In keeping with these findings we observed no genotype-specific differences in the number of freshly isolated human neutrophils migrating through a HUVEC layer in response to MIP-2 (figure 4B). As the transmigration assays were performed under static conditions, we adopted a previously described peritonitis model [25] to evaluate the behaviour of the cells *in vivo*. The *in vitro* differentiated PMN cell lines expressing the 77H or 77R CD11b variant were labelled with DDAO or CFSE and co-injected i.v. at a 1∶1 ratio into wild type C57BL/6 mice. Immediately after the adoptive transfer, inflammation was induced in the peritoneum either with thioglycollate or MIP-2 injected i.p. and the number of DDAO- and CFSE-labelled neutrophils elicited into the peritoneum assessed by flow cytometry at different time points. Using this *in vivo* migration assay we found that the same number of (mCD18/hCD11b-77R)- and (mCD18/hCD11b-77H)-expressing PMNs accumulated in the peritoneum irrespective of the inflammatory stimuli applied (figure 4C, 4D). As predicted the presence of the hybrid CR3 molecule, regardless of the CD11b variant transduced, decreased the number of PMNs in the peritoneum compared to that of the PMN cell line lacking CD11b (data not shown) confirming that an interaction between the hybrid mCD18/hCD11b molecule and mouse endothelium had occurred. No labelled neutrophils were recovered from the peritoneal lavage of animals injected with PBS. We also explored other typical PMN functions: R77H polymorphism failed to alter the oxidative burst and netosis of PMNs after stimulation with PMA (data not shown).

### Modulation of cytokine secretion

**Figure 5:**
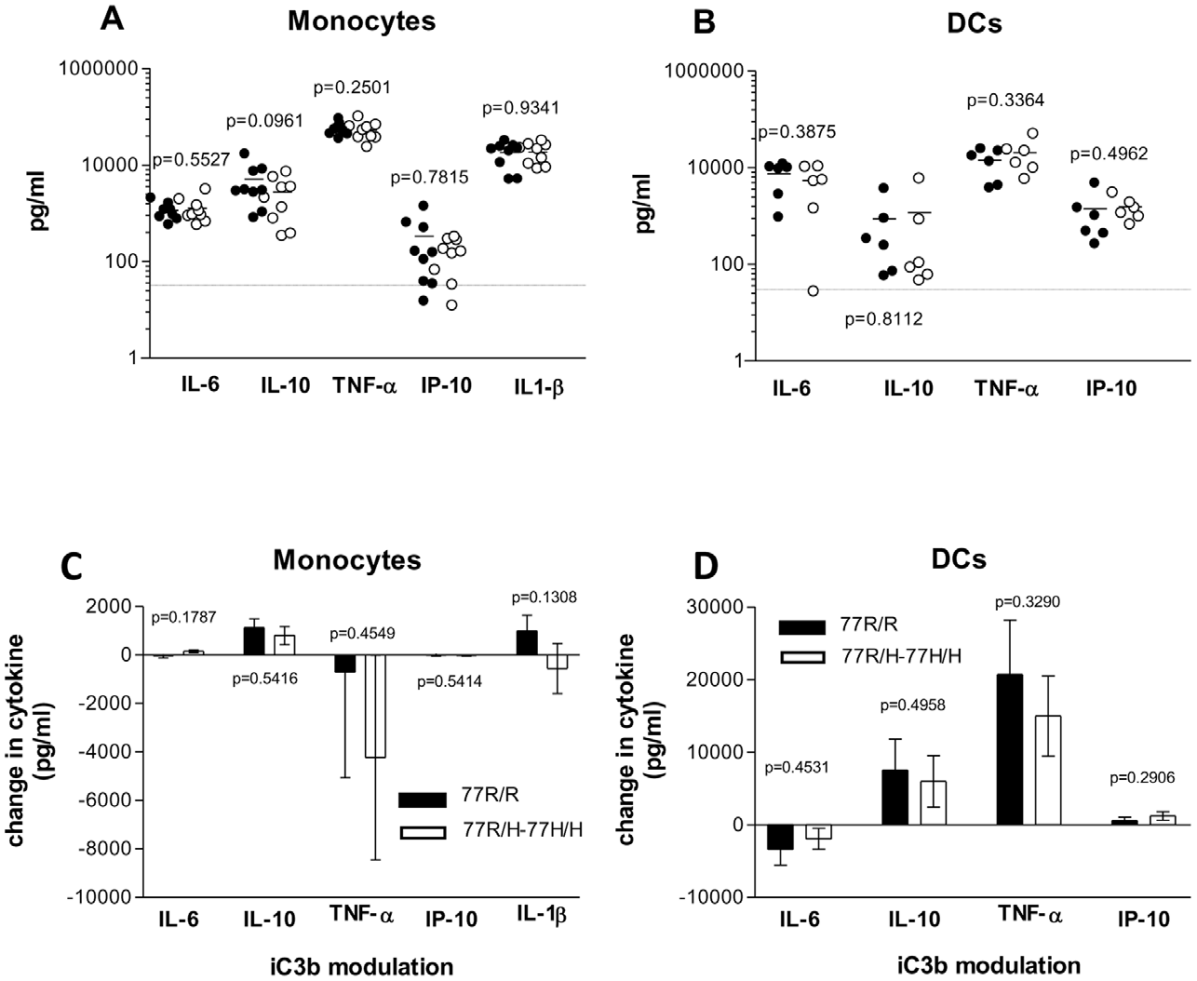
Cytokine response. Monocytes (A), DCs (B) were stimulated with 2 µg/ml and 10 µg/ml of TLR7/8 ligand (R848) respectively for 24 h. Cytokines quantified using a bead multiplex assay. Closed symbols: 77R/R cells, open symbols: 77R/H-77H/H cells. Each dot represents a single individual, bars denote means. No significant differences between the two CD11b genotypes. Statistical analysis by paired t test. (C, D) Modulation of TLR7/8-induced cytokine release by hiC3b-coated beads. Monocytes (C), DCs (D) were fed with hiC3b-coated beads one hour prior to R848 stimulation. The cytokine changes between the samples with and without CR3 pre-engagement with iC3b are shown with the p values indicated. Data are expressed as mean+/−SEM. The cytokine responses of 77R/R cells (black column) and 77R/H-77H/H cells (white columns) were not statistically different in paired assays. IL, interleukin; TNF-α, tumour necrosis factor alpha; IP-10, Interferon gamma-induced protein 10.

There is evidence in the literature that CR3 can alter the cytokine production by TLR-stimulated monocytes/macrophages and DCs [31]–[35]. More recently the R77H CD11b polymorphism has been shown to induce a different inhibitor effect on the TLR7/8-mediated pro-inflammatory cytokine release by monocytes [18]. We initially measured the cytokine secretion (IL-6, IL-10, TNF-α, IP-10 and IL-1β) by monocytes and DCs after TLR7/8 stimulation. The CD11b genotype did not influence the cytokine response (figure 5A and 5B). We also analysed the cytokine production after overnight stimulation with beads or hiC3b-coated beads. Neither of them induced detectable cytokines demonstrating no endotoxin contamination (data not shown). We then investigated whether the 77R/H monocytes and DCs down-regulated less efficiently the pro-inflammatory response induced by TLR7/8 activation as recently reported [18]. To this end, we compared the cytokine production with/without pre-incubation with hiC3b-coated beads and analysed the difference between the two samples. In DCs the pre-incubation with hiC3b-coated beads induced a strong up-regulation of IL-10 and TNF-α with a modest down-modulation of IL-6 (figure 5D). The effect on IP-10 was barely detectable. In monocytes, the pre-incubation with hiC3b-coated particles resulted in a small increase of IL-10, a decrease in TNF-α, whilst IL-6 and IP-10 remained largely unchanged. The changes in IL-1β secretion were variable (figure 5C). More importantly, in both cell types we found no statistically significant differences between the two CD11b variants in the cytokine modulation by iC3b-coated particles after TLR7/8 stimulation. In selected experiments we also used iC3b-gRBCs to mirror more closely the experimental conditions used by Rhodes et al [18]. Although we observed a slightly different cytokine pattern with a stronger effect on IL-1β secretion, we failed to identify a genotype-specific difference in monocyte cytokine responses (figure S4). Similarly CR3 ligation by hiC3b-coated particles modified the TLR9-mediated pro-inflammatory cytokine released by monocytes but the effect was not modulated by the CD11b genotype (data not shown).

## Discussion

Several genes that exert their effect through relatively common allelic variants have been shown to be associated with SLE by GWA studies [9], [10], [36]–[41] One of most prominent susceptibility factors for SLE was found to be a SNP (rs1143679) which confers the R77H amino acid substitution in CD11b. The functional effects of this variant remains elusive. By using a broad approach (*ex-vivo* human cells and immortalized PMN and macrophage lines expressing the hCD11b variants) and by analysing the main human CR3 expressing cells (monocytes, neutrophils, macrophages and DCs) herein we demonstrate that in these cells the lupus susceptibility allele (77H) impairs only the uptake of hiC3b-coated targets without altering other CR3-mediated functions including TLR7/8-induced cytokine secretion and neutrophil extravasation. These findings highlight the importance of minor functional changes in genes controlling waste disposal mechanisms in lupus pathogenesis.

Two recent reports have suggested that the CD11b lupus risk allele causes an impaired adhesion/phagocytosis and abnormal cytokine modulation after TLR7/8 stimulation in monocytes [17], [18]. In both studies the function of the risk allele was compared to the non-risk allele using transfected cell lines or homozygous individuals. However, as the lupus risk allele has a frequency of only 9–11% in populations of European and African descent [9]–[11], with a much lower frequency (<0.01) in Asian Populations [39], the vast majority of the SLE patients are heterozygous for the rs1143679 SNP: approximately 27% are 77R/H compared to ∼3% 77H/H. Assuming a codominant model the odds ratio was significantly increased for both the 77R/H (1.63) and the 77H/H (4.64) genotypes [11]. Therefore in our study we have deliberately elected to analyse healthy 77R/H individuals as they mirror more closely the disease population. In all the assays with *ex-vivo* human cells we have also included in the analysis 3 homozygous 77H/H individuals whose cells consistently behaved like the 77R/H cells showing that a single 77H allele was sufficient to induce the cellular abnormalities detected. Therefore, though we recognise that by adopting this approach some subtle differences between the two CD11b variants might have been overlooked, we think that our data mirror more realistically the modest magnitude of risk carried by the 77H Cd11b variant.

It has been postulated that lupus patients might have higher surface expression of CD11b resulting in an increased cellular infiltrate and amplified inflammation [41]–[43]. Consistent with the findings reported by Rhodes et al [18] we failed to detect any obvious difference between 77R/R and 77R/H-77H/H individuals in the surface expression of CD11b including its active form assessed by the CBRM1/5 antibody.

The position of the 77R/H polymorphism in the beta-propeller domain predicts that it is unlikely to affect the iC3b binding site [11], [44]. In keeping with this prediction we found no defects in the binding of 77H PMNs to iC3b-RBC in the rosetting assay [15], [18]. We observed, however, a significant reduction in the uptake of iC3b-coated targets (gRBC and beads) by neutrophils indicating that phagocytosis is not simply a function of ligand/receptor interaction, but entails several subsequent events in which CR3 may play a role. Not surprisingly the impaired phagocytosis was also detected in 77R/H monocytes and macrophages but not in immature DCs. We confirmed the specificity of the effect for the 77H variant using stably genetically-modified *in vitro*-derived macrophage lines lacking CD11b or expressing variant-specific CD11b molecules. Using the allele-specific macrophage cell lines we were able to show that the lupus-associated 77H allele impaired also the phagocytosis of iC3b-coated apoptotic cells, a defect seen in macrophages from lupus patients [45], [46]. Overall our data not only provide further support to previous reports linking the 77H variant to impaired phagocytosis [17], [18] but significantly expand previous observations by demonstrating that the impaired phagocytosis associated with the 77H CD11b variant is not macrophage-restricted.

CR3 is known to bind a large range of ligands and to contribute to leukocyte extravasation. Previous reports [17], [18] have shown that the lupus CD11b allele reduces cell adhesion to several CR3 ligands, including ICAM-1, under static and/or flow conditions. However, these data, obtained in part with transfected cells [17], are hard to reconcile with the clinical observations of hypercellularity present in injured tissue. To circumvent some of the limitations of the previous assays we applied a novel *in vivo* adoptive transfer approach [25]. We found no genotype-specific difference in the migration of PMNs under two distinct inflammatory stimuli casting doubts on some of the previous findings [17], [18] and demonstrating that the 77H allele is unlikely to affect this CR3-mediated pathway.

There is a growing body of evidence demonstrating that engagement of CD11b can mediate both positive and negative regulation of TLR signalling with contradictory results accordingly to the experimental conditions applied [31], [32], [34], [47]–[49]. Under our experimental conditions we could not replicate the genotype-specific difference in the cytokine responses induced by TLR7/8 stimulation after CR3 ligation recently reported by Rhodes et al. in monocytes [18]. In our assay pre-incubation of monocytes with hiC3b-coated beads had a less striking effect on the TLR7/8-induced cytokine release compared to that reported [18] with just an appreciable, though variable, inhibitory effect on TNF-α production. However, regardless of the degree of cytokine inhibition, the 77H variant did not affect the cytokine response. Though we recognise that the use of 77R/H cells could have masked a potential difference, most likely the explanation for the discrepancy between the studies lies in the different hiC3b-coated targets used. We deliberately avoided using sheep RBCs for two main reasons: i) sheep RBCs require opsonisation with rabbit anti-sheep erythrocyte IgM and human serum, both potential source of contaminants causing non-specific interactions/effects; ii) RBCs can release several molecules including haemoglobin that is known to bind to CD163 on monocytes/macrophages dampening the inflammatory response [50]–[52]. Consistent with the explanation that, at least to a certain degree, the CR3-mediated inhibitor effect on the TLR7/8-induced pro-inflammatory cytokine was due to the use of the RBCs, we observed a much stronger IL-1β inhibition when we pre-incubated the monocytes with gRBC-iC3b. However, we used guinea pig red blood cells that do not require opsonisation with IgM for complement activation and our cytokine pattern did not entirely replicate the one reported by Rhodes et al. Interestingly in DCs we observed a marked synergistic positive effect on IL-10 and TNF-α secretion that was again comparable between 77R/R and 77R/H cells. Therefore CR3 may well act as an important regulator of several TLR signalling pathways including the TLR7/8 but the effect does not appears to be influenced by the R77H amino acid substitution.

In summary our observations demonstrate that the CD11b lupus-associated variant leads to an impaired phagocytosis of complement-opsonised targets, including apoptotic cells, by monocytes/macrophages and PMNs. A reduced clearance of iC3b-coated targets might lead to increased tissue damage and inflammation, providing a plausible explanation for the genetic linkage identified by GWA studies. CR3 may also interact with the Fc receptors on the surface of neutrophils/macrophages modulating the effect of immune complex binding and this cross-talk warrants further analysis. However, one should not ignore the fact that CR3 is also expressed on NK and a subset of B cells, cell types that have recently shown to contribute to SLE pathogenesis [53]–[55] and the effect of the lupus CD11b allele on these cells remains to be examined.

## Supporting Information

Figure S1 **Fold increase expression of CD11b on PMNs after PMA stimulation.** Cell surface expression of CD11b was assessed by flow cytometry using two antibodies: ICRF44 and CBRM1/5 (active state). Closed symbols indicate the 77R/R donors, open symbols the 77R/H-77H/H donors. Data were normalised to the MFI of the respective unstimulated PMN. Pooled results from 3 experiments are presented as mean of fold increase ± SEM.
(TIF)

Figure S2 **Phagocytosis of gRBCs-miC3b.** Uptake by macrophages (A) and PMNs (B) carrying one of the two CD11b variants (77R/R closed columns, 77R/H-77H/H open columns). gRBCs were labelled with pHrodo and the uptake was quantified by flow cytometry. The data are represented as percentage of phagocytosis (macrophages:18 pairs; PMN: 13 pairs). Data are expressed as mean+/−SEM. Statistical analysis by paired T test.
(TIF)

Figure S3 **Assays with** ***Itgam*** **^−/−^ cell lines reconstituted with a hybrid CR3 molecule (mCD18/hCD11b-77R or mCD18/hCD11b-77H).** (A) Cell-surface expression of the hybrid molecules quantified by flow cytometry using an anti-human CD11b antibody (ICRF44). Data are presented for macrophages and PMNs. Dotted line: CD11b-deficient cells transduced with an empty vector; solid line: mCD18/hCD11b-77R cells; dashed line: mCD18/hCD11b-77H; shaded histogram: cells derived from wild type C57BL/6 mice. (B) Engulfment of pHrodo-loaded gRBC opsonised with miC3b. The percentage of phagocytosis was determined by flow cytometry at one hour time point. A significant difference between the two macrophage lines expressing the hybrid CR3 molecule was detected (p = 0.0256). The parental CD11b-deficient macrophages were used as negative control to confirm the specificity for CR3 of the assay. Bars indicate means. (C) Phagocytosis of opsonised (black column) or non opsonised (empty column) pHrodo labelled murine apoptotic thymocytes. The percentage of phagocytosis was determined by flow cytometry at one hour time point. Pooled data of 5 independent experiments. Data were normalised to the C57BL/6 cell line and are expressed as mean+/−SEM. Statistical analysis by paired T test. (D) Rosetting assay with CFSE-labelled gRBC-hiC3b. Percentage of neutrophils bound to CFSE-labelled gRBCs was measured by flow cytometry. Square symbols: mCD18/hCD11b-77R; triangle symbols: mCD18/hCD11b-77H; circle symbols: wild type C57BL/6 neutrophils.
(TIF)

Figure S4 **Cytokine response.** Modulation of TLR7/8-induced cytokine release by iC3b-gRBC. Monocytes were fed with iC3b-gRBC or gRBC one hour prior to R848 stimulation. The cytokine changes between the samples with and without CR3 pre-engagement are shown with the p values indicated. Data are expressed as mean+/−SEM. The cytokine responses of 77R/R cells (black column) and 77R/H-77H/H cells (white columns) were not statistically different in paired assays. IL, interleukin; TNF-α, tumour necrosis factor alpha; IP-10, Interferon gamma-induced protein 10.
(TIF)
