# Supplementary figures and images for: Phagocytosis Is the Main CR3-Mediated Function Affected by the Lupus-Associated Variant of CD11b in Human Myeloid Cells
Source: PLoS One. 2013 Feb 22;8(2):e57082. doi: 10.1371/journal.pone.0057082 (PMC3579793; doi:10.1371/journal.pone.0057082)

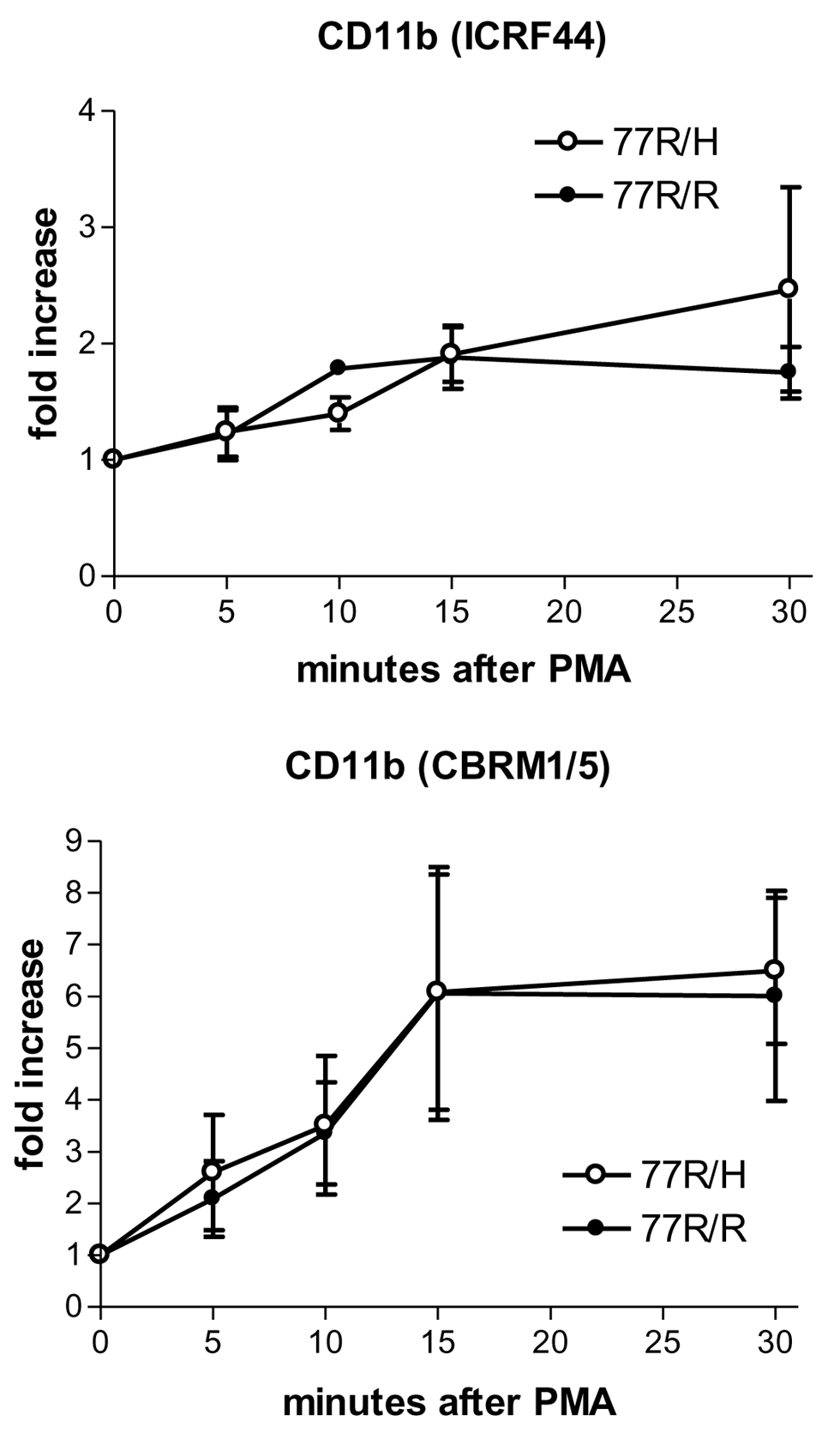

Supplement: Figure S1 — Fold increase expression of CD11b on PMNs after PMA stimulation. Cell surface expression of CD11b was assessed by flow cytometry using two antibodies: ICRF44 and CBRM1/5 (active state). Closed symbols indicate the 77R/R donors, open symbols the 77R/H-77H/H donors. Data were normalised to the MFI of the respective unstimulated PMN. Pooled results from 3 experiments are presented as mean of fold increase ± SEM. (TIF) [file pone.0057082.s001.tif]

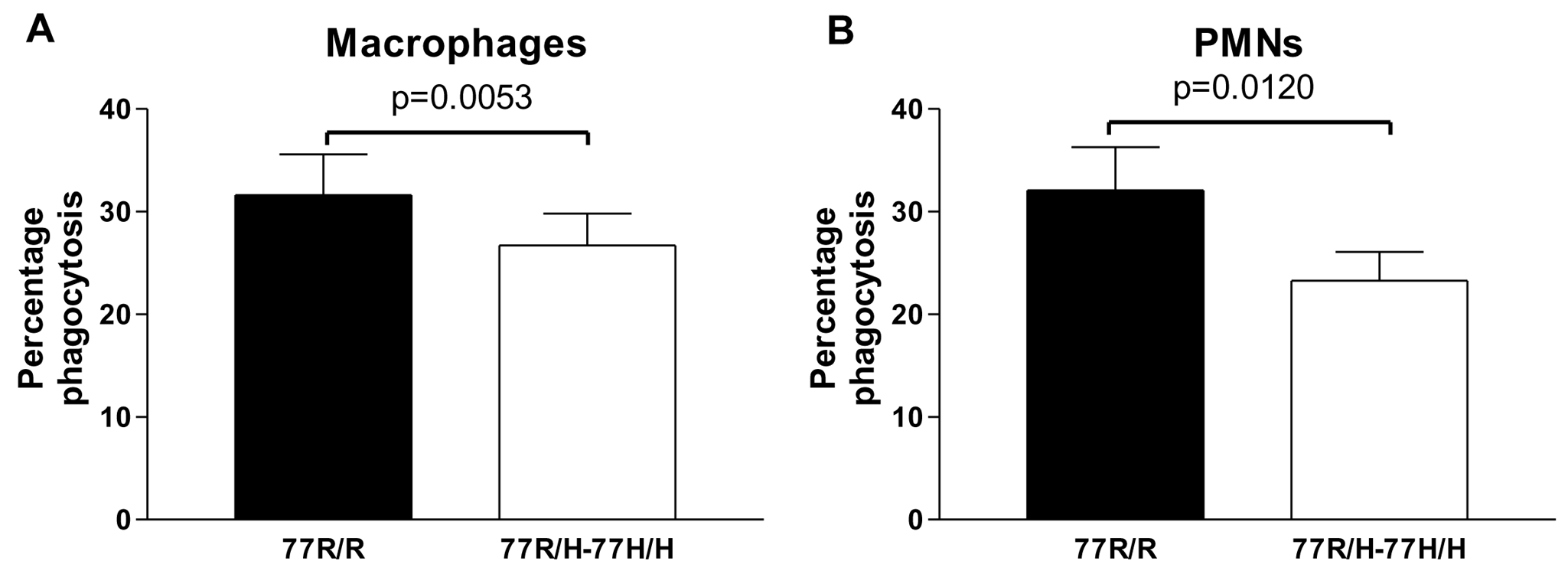

Supplement: Figure S2 — Phagocytosis of gRBCs-miC3b. Uptake by macrophages (A) and PMNs (B) carrying one of the two CD11b variants (77R/R closed columns, 77R/H-77H/H open columns). gRBCs were labelled with pHrodo and the uptake was quantified by flow cytometry. The data are represented as percentage of phagocytosis (macrophages:18 pairs; PMN: 13 pairs). Data are expressed as mean+/−SEM. Statistical analysis by paired T test. (TIF) [file pone.0057082.s002.tif]

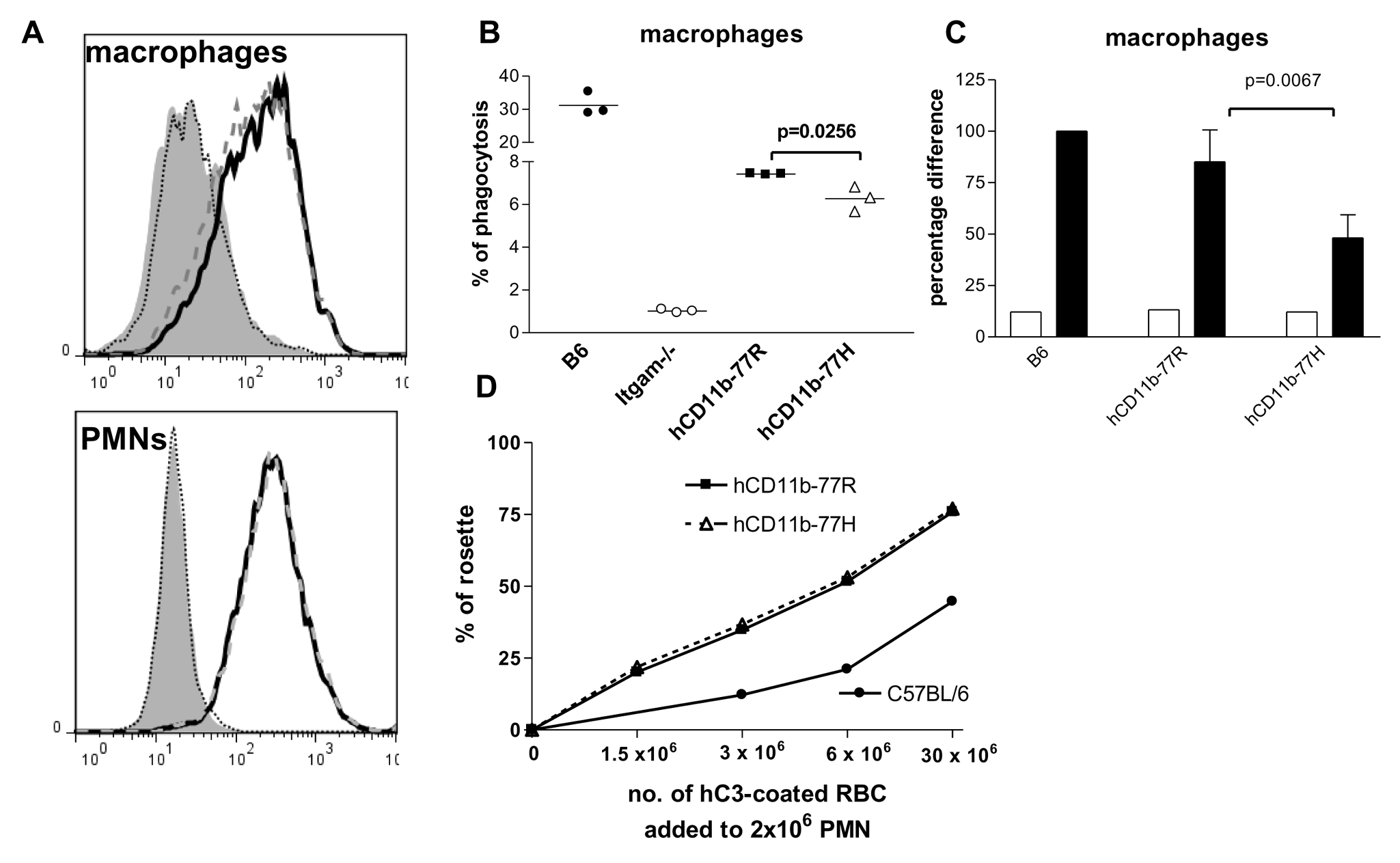

Supplement: Figure S3 — Assays with Itgam −/− cell lines reconstituted with a hybrid CR3 molecule (mCD18/hCD11b-77R or mCD18/hCD11b-77H). (A) Cell-surface expression of the hybrid molecules quantified by flow cytometry using an anti-human CD11b antibody (ICRF44). Data are presented for macrophages and PMNs. Dotted line: CD11b-deficient cells transduced with an empty vector; solid line: mCD18/hCD11b-77R cells; dashed line: mCD18/hCD11b-77H; shaded histogram: cells derived from wild type C57BL/6 mice. (B) Engulfment of pHrodo-loaded gRBC opsonised with miC3b. The percentage of phagocytosis was determined by flow cytometry at one hour time point. A significant difference between the two macrophage lines expressing the hybrid CR3 molecule was detected (p = 0.0256). The parental CD11b-deficient macrophages were used as negative control to confirm the specificity for CR3 of the assay. Bars indicate means. (C) Phagocytosis of opsonised (black column) or non opsonised (empty column) pHrodo labelled murine apoptotic thymocytes. The percentage of phagocytosis was determined by flow cytometry at one hour time point. Pooled data of 5 independent experiments. Data were normalised to the C57BL/6 cell line and are expressed as mean+/−SEM. Statistical analysis by paired T test. (D) Rosetting assay with CFSE-labelled gRBC-hiC3b. Percentage of neutrophils bound to CFSE-labelled gRBCs was measured by flow cytometry. Square symbols: mCD18/hCD11b-77R; triangle symbols: mCD18/hCD11b-77H; circle symbols: wild type C57BL/6 neutrophils. (TIF) [file pone.0057082.s003.tif]

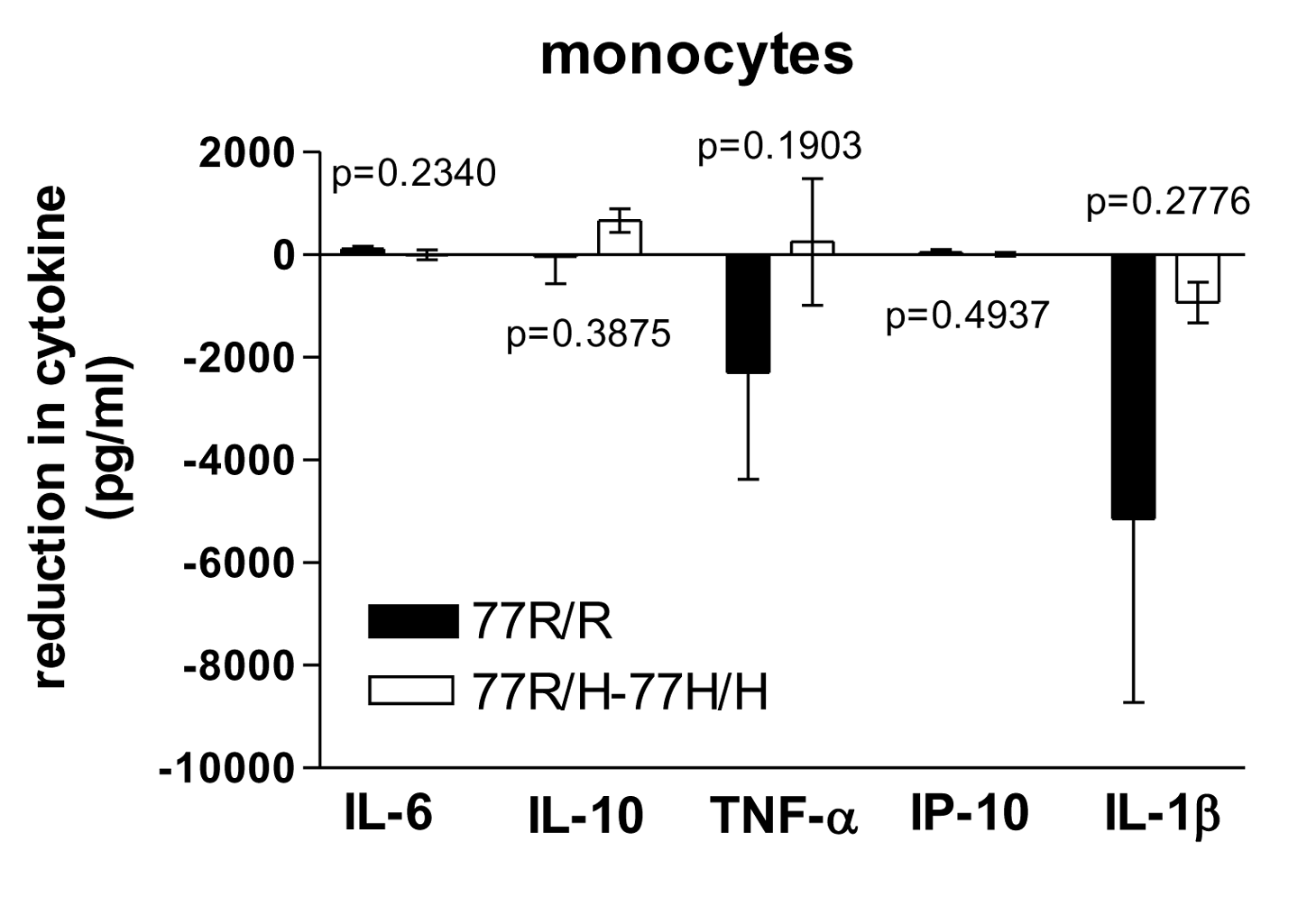

Supplement: Figure S4 — Cytokine response. Modulation of TLR7/8-induced cytokine release by iC3b-gRBC. Monocytes were fed with iC3b-gRBC or gRBC one hour prior to R848 stimulation. The cytokine changes between the samples with and without CR3 pre-engagement are shown with the p values indicated. Data are expressed as mean+/−SEM. The cytokine responses of 77R/R cells (black column) and 77R/H-77H/H cells (white columns) were not statistically different in paired assays. IL, interleukin; TNF-α, tumour necrosis factor alpha; IP-10, Interferon gamma-induced protein 10. (TIF) [file pone.0057082.s004.tif]
